# Incorporation of Synthetic mRNA in Injectable Chitosan-Alginate Hybrid Hydrogels for Local and Sustained Expression of Exogenous Proteins in Cells

**DOI:** 10.3390/ijms19051313

**Published:** 2018-04-27

**Authors:** Heidrun Steinle, Tudor-Mihai Ionescu, Selina Schenk, Sonia Golombek, Silju-John Kunnakattu, Melek Tutku Özbek, Christian Schlensak, Hans Peter Wendel, Meltem Avci-Adali

**Affiliations:** Department of Thoracic and Cardiovascular Surgery, University Hospital Tuebingen, Calwerstraße 7/1, 72076 Tuebingen, Germany; heidi.steinle@googlemail.com (H.S.); tudor_mihai.ionescu@yahoo.de (T.-M.I.); schenkse@hs-albsig.de (S.S.); sonia.golombek@klinikum.uni-tuebingen.de (S.G.); silju_j1984@yahoo.de (S.-J.K.); mtutkuozbek@gmail.com (M.T.O.); Christian.Schlensak@med.uni-tuebingen.de (C.S.); hans-peter.wendel@med.uni-tuebingen.de (H.P.W.)

**Keywords:** synthetic mRNA, injectable hydrogels, mRNA delivery, production of exogenous proteins

## Abstract

The application of synthetic messenger RNA (mRNA) exhibits various advantages, such as expression of desired proteins in cells without genomic integration. In the field of tissue engineering, synthetic mRNAs could be also used to modulate the protein expression in implanted cells. Therefore, in this study, we incorporated synthetic humanized Gaussia luciferase (hGLuc) mRNA into alginate, chitosan, or chitosan-alginate hybrid hydrogels and analyzed the release of hGLuc mRNA from these hydrogels. After 3 weeks, 79% of the incorporated mRNA was released from alginate hydrogels, approximately 42% was released from chitosan hydrogels, and about 70% was released from chitosan-alginate hydrogels. Due to the injectability, chitosan-alginate hybrid hydrogels were selected for further investigation of the bioactivity of embedded hGLuc mRNA and the stability of these hydrogels was examined after the incorporation of synthetic mRNA by rheometric analysis. Therefore, HEK293 cells were incorporated into chitosan-alginate hydrogels containing mRNA transfection complexes and the luciferase activity in the supernatants was detected for up to 3 weeks. These results showed that the biodegradable chitosan-alginate hybrid hydrogels are promising delivery systems for sustained delivery of synthetic mRNAs into cells. Since chitosan-alginate hybrid hydrogels are injectable, the hydrogels can be simultaneously loaded with cells and the desired synthetic mRNA for exogenous protein synthesis and can be administered by minimally invasive local injection for tissue engineering applications.

## 1. Introduction

In recent years, the application of synthetic messenger RNAs (mRNAs) for the production of therapeutic proteins has gained interest due to their advantageous properties over the use of DNA-based methods, including a safe, easy, and efficient translation of proteins [1,2]. The synthetic mRNA does not integrate into the host genome, it does not need to enter the nucleus for the translation of proteins, and after reaching the cytosol, delivered mRNA is immediately translated by ribosomes into proteins. So far, several applications with therapeutic mRNAs have been investigated, including vaccination, treatment of cancer, infectious diseases, protein deficiency disorders, inflammation, as well as production of growth factors, cellular reprogramming and differentiation [3,4,5,6,7,8,9,10,11,12,13,14,15,16,17,18].

Hydrogels are three-dimensional (3D) networks composed of natural or synthetic hydrophilic polymer chains, which can be cross-linked physically or chemically [19,20,21]. Especially, due to their high biocompatibility, biodegradability, and low toxicity, natural polymers are beneficial compared to synthetic polymers [19,22]. Thus, hydrogels have attracted increased attention especially in tissue engineering applications and regenerative medicine, e.g., for the regeneration and repair of tissues or the controlled release of drugs [20,23,24]. Multiple studies demonstrated the applicability of hydrogels for the release of a broad range of different therapeutic agents, such as growth factors [25,26,27,28,29], insulin [30,31,32], and anti-cancer drugs [27,33,34,35,36].

Alginate and chitosan are very frequently used biomaterials for the generation of hydrogels. Alginate is a polyanionic polysaccharide, which consists of alternating blocks of (1–4)-linked β-d-mannuronic acid (M) and α-l-glucoronic acid (G) monomers [37]. The polymers can be ionically crosslinked using bivalent cations, such as Ca^2+^, to obtain a three-dimensional network [38]. Alginate solutions can also be injected in vivo and hydrogels can then be generated by Ca^2+^ ions that are present in the surrounding tissue [39]. Furthermore, due to their similarity to extracellular matrix proteins, alginate hydrogels have been used as a carrier for cells, and have proven to promote the release of nucleic acids, such as small interfering RNA (siRNA) and plasmid DNA (pDNA) [40,41,42,43]. In contrast to alginate, chitosan is a cationic polysaccharide and it is produced by the deacetylation of chitin. The gelation of chitosan can be performed by adding glycerol phosphate (GP). Thereby, injectable chitosan solutions can be generated, which are thermo-responsive at physical pH and enable in situ formation of gels upon warming to body temperature [44]. Due to their charge, cationic polymers are favorable for delivery of anionic molecules. Thus, chitosan is able to condense nucleic acids into cationic polyplexes and thereby, facilitate transfection [45]. The release of functional nucleic acids from chitosan hydrogels was demonstrated for siRNA, which resulted in a prolonged and localized gene silencing, and for pDNA, which promoted tissue regeneration [43,45,46,47].

Chitosan and alginate are known to form polyelectrolyte complexes and have been combined to create hybrid hydrogels [48,49]. Due to electrostatic interactions of the opposite charged polymers, this hybrid material combination demonstrates an increased stability compared to both polymers alone [50]. In previous studies, chitosan-alginate hybrid hydrogels supported cell growth, wound healing, bone regeneration, cartilage tissue engineering, and they were used for the treatment of myocardial infarction and to induce angiogenesis [49,51,52,53,54]. Furthermore, cationic pDNA-nanoparticles incorporated into a 3D chitosan-alginate porous scaffold demonstrated gene delivery in prostate cancer cells in vitro and in vivo [55].

Drug-releasing systems continuously delivering synthetic mRNA locally to the cells during the required period could represent a suitable alternative to repeated administration of synthetic mRNA, especially for in vivo applications. To obtain a sustained expression of an exogenous protein, we developed a local delivery approach based on mRNA embedded injectable hydrogels. Here, we analyzed for the first time, the applicability of synthetic mRNA in hydrogels for the extended transfection of cells incorporated into hydrogels. Therefore, synthetic secretable humanized Gaussia luciferase (hGLuc) mRNA was loaded into alginate, chitosan, or chitosan-alginate hybrid hydrogels and the release characteristics of synthetic mRNA was investigated over a period of 21 days. Afterwards, the bioactivity of incorporated mRNA and the transfectability of cells were determined.

## 2. Results

### 2.1. Release of Cy3-Labeled hGLuc mRNA from Hydrogels

**Figure 1:**
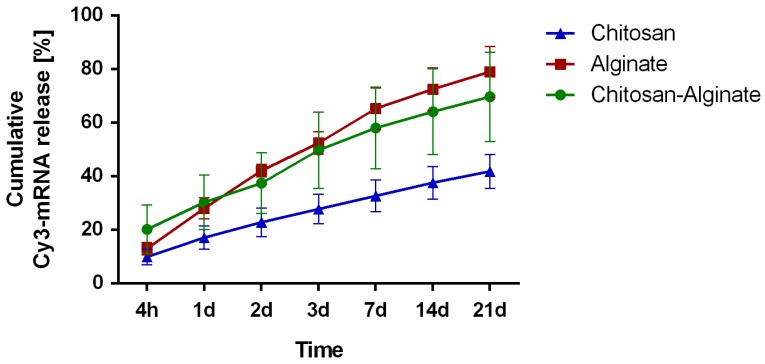
Cumulative release profile of Cy3-labeled hGLuc mRNA from alginate, chitosan, and chitosan-alginate hybrid hydrogels. Hydrogels consisting of alginate (1.3% alginate and 19.9 mM CaCl_2_), chitosan (1.9% chitosan, 4.2% glycerol phosphate (GP)), or chitosan-alginate (1.4% chitosan, 4% GP, and 0.6% alginate) were prepared and loaded with 4 µg of Cy3-hGLuc mRNA transfection complexes. The release of Cy3-mRNA was determined after 4 h, 1, 2, 3, 7, 14, and 21 days (d). The total amount of released mRNA after 21 days was 79% for alginate, 42% for chitosan, and 70% for chitosan-alginate hybrid hydrogels. Data are shown as mean ± SEM (*n* = 3).

In order to investigate the release kinetics of synthetic mRNA from alginate, chitosan, and chitosan-alginate hybrid hydrogels, 4 µg of complexed Cy3-labeled hGLuc mRNA was incorporated into the hydrogels. After 4 h, 1, 2, 3, 7, 14, and 21 days of incubation at 37 °C, the relative fluorescence intensity (RFU) of Cy3-labeled mRNA was measured in 50 µL supernatant in triplicates. The amount of released mRNA was calculated by using a standard curve ranging from 31 to 1000 ng Cy3-labeled mRNA. According to the measurements, all hydrogels demonstrated a sustained release of Cy3-labeled hGLuc mRNA over 21 days (Figure 1). At 21 days, alginate hydrogels (1.3% alginate, 19.9 mM CaCl_2_) released 79.0% of the incorporated Cy3-mRNA. Chitosan hydrogels (1.9% chitosan, 4.2% GP) showed compared to alginate hydrogels a slower mRNA release and only 41.8% of the incorporated mRNA was released during 21 days. The incorporation of mRNA into a hybrid hydrogel containing 1.4% chitosan, 0.6% alginate and 4% GP resulted in slower release compared to alginate hydrogels. After 21 days of incubation, 69.6% of the incorporated Cy3-mRNA was released.

### 2.2. Rheological Characterization of mRNA-Loaded Chitosan-Alginate Hydrogels

Due to the injectability of chitosan-alginate hydrogels compared to chitosan hydrogels and slower mRNA release compared to alginate hydrogels, chitosan-alginate hydrogels were selected for further analysis. To assess hydrogel characteristics and the stability of hydrogels over several days in vitro, chitosan-alginate hydrogels were loaded without or with 4 µg of complexed hGLuc mRNA. As a control, hydrogels with transfection reagent were generated. Rheological measurements were performed directly after gelation (day 0) and after the incubation of hydrogels with 0.5 mL Dulbecco’s phosphate-buffered saline (DPBS) for 1, 7, 14, and 21 days at 37 °C.

**Figure 2:**
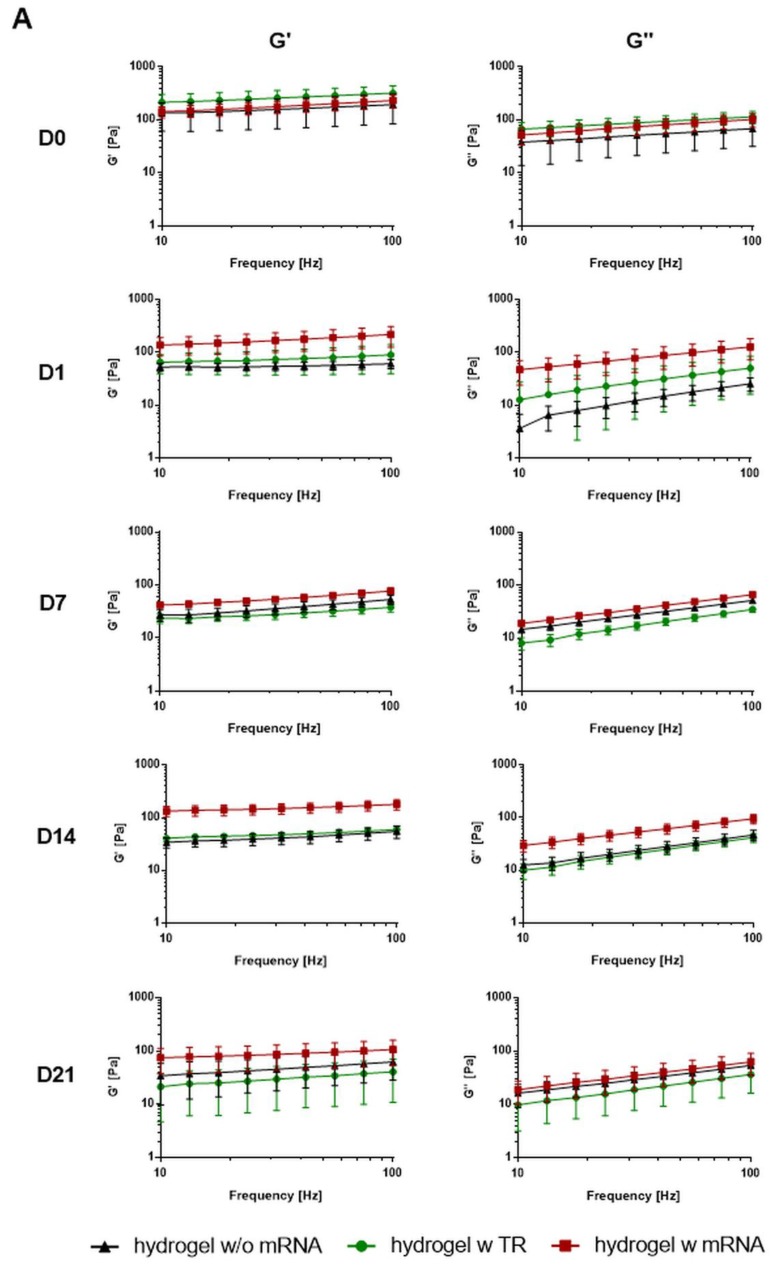

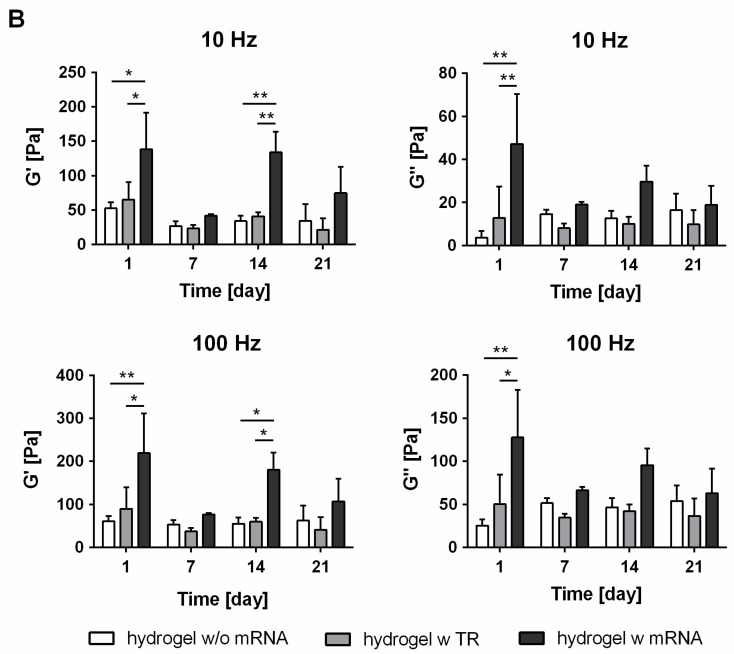
Rheological characterization of mRNA containing chitosan-alginate hybrid hydrogels over time. (**A**) Hydrogels without and with 4 µg mRNA and hydrogels containing only the transfection reagent (TR) were generated. Storage (G′) and loss moduli (G′′) were analyzed without adding Dulbecco’s phosphate-buffered saline (DPBS) (D0) and after the addition of DPBS and incubation for 1 (D1), 7 (D7), 14 (D14), and 21 (D21) days using oscillatory squeeze-flow rheometer with a frequency scan of 10–100 Hz. Hydrogels without and with mRNA showed higher G′ than G′′ modulus after different time points of incubation in DPBS, which indicates stable gel-like properties over time; (**B**) Statistical analysis of G′ and G′′ values at 10 and 100 Hz. Results are shown as mean ± SD (*n* = 3). Statistical differences were determined using repeated measures two-way ANOVA with Bonferroni’s multiple comparisons test. (* *p* < 0.05, ** *p* < 0.01).

Over the whole frequency range (10–100 Hz) and incubation time, the hydrogels demonstrated higher storage (G′) modulus compared to the loss (G′′) modulus, which indicated stable gel-like properties of the chitosan-alginate hydrogels (Figure 2). After the addition of DPBS to the gels (day 1), mRNA-loaded chitosan-alginate hydrogels showed significantly higher elastic G′ and viscous G′′ values (187 ± 79 Pa G′ and 185 ± 86 Pa G′′) compared to hydrogels without mRNA (54 ± 19 Pa G′ and 38 ± 16 Pa G′′), representing higher mechanical strength and stiffness of the mRNA-loaded hydrogel. Although after 7, 14, and 21 days, the mRNA containing hydrogels showed higher G′ values than the hydrogels without mRNA, only the value after 14 days of incubation was statistically significant (Figure 2B). A slight decrease of G′ and G′′ was detected in all hydrogels from day 1 to day 7, which could be the result of hydrogel degradation.

### 2.3. Bioactivity of hGLuc mRNA Incorporated into Chitosan-Alginate Hydrogels

**Figure 3:**
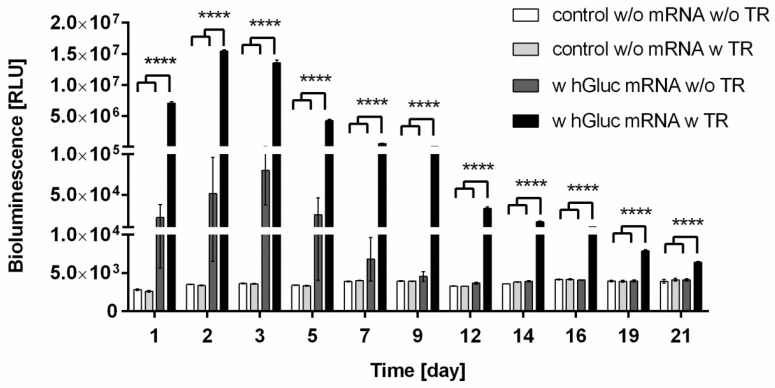
Bioactivity of hGLuc mRNA incorporated into chitosan-alginate hybrid hydrogels. Chitosan-alginate hydrogels with 4 µg hGLuc mRNA complexed with or without transfection reagent (TR) were generated and 1.5 × 10^5^ HEK293 cells were incorporated. Additionally, hydrogels containing HEK293 cells without mRNA and TR or with TR were produced. The production of hGLuc was determined from 1 to 21 days in medium using luciferase assay. Data are shown as mean ± SEM, (*n* = 4). Statistical differences were determined using one-way ANOVA with Bonferroni’s multiple comparisons test. (**** *p* < 0.0001).

Chitosan-alginate hydrogels containing 1.5 × 10^5^ HEK293 cells were generated and 4 µg hGLuc mRNA with or without complexation with transfection reagent was incorporated into the hydrogels. Additionally, hydrogels without mRNA and transfection reagent or only with transfection reagent were produced as negative controls. The ability of hGLuc mRNA to mediate protein expression in cells was analyzed over a period of 21 days by detection of luciferase activity in the supernatant (Figure 3). During 21 days, significantly increased amount of hGLuc was detected in hydrogels containing synthetic hGLuc mRNA transfection complexes compared to control hydrogels. The highest luciferase activity was measured after 48 h of incubation. Afterwards, a continuous decrease of luciferase activity was determined. Due to the ability of chitosan to generate complexes with negatively charged oligonucleotides, we further investigated if the HEK293 cells can be transfected without using the transfection reagent. Although hGLuc protein could be detected until 7 days, the measured values showed large fluctuations and the differences were statistically not significant. Clearly, less protein was produced when the mRNA was incorporated without transfection reagent compared to the hydrogels with complexed mRNA (Figure 3). After 9 days, the protein expression dropped to the levels of control hydrogels without mRNA. These results demonstrated that the generation of mRNA transfection complexes are required to efficiently transfect cells and to continuously produce a desired protein in cells. The hGLuc expression of cells after a single transfection with 4 µg hGLuc mRNA over 4 days is shown in Supplementary Figure S1.

### 2.4. Influence of hGLuc mRNA Loading into Chitosan-Alginate Hybrid Hydrogels on Cell Viability

**Figure 4:**
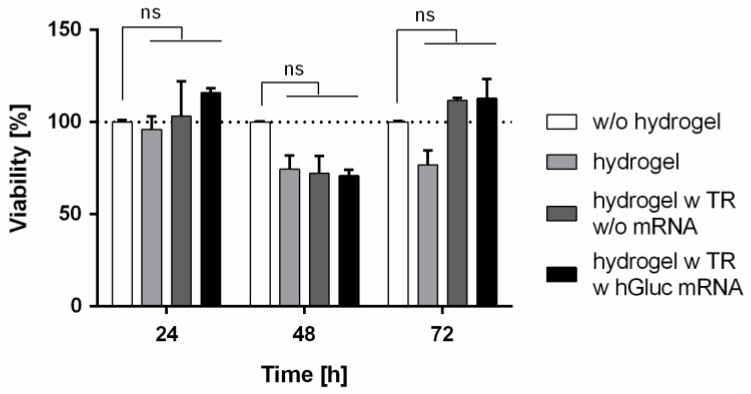
Analysis of the influence of hGLuc mRNA loading into chitosan-alginate hybrid hydrogels on cell viability using PrestoBlue^®^ assay. 1.5 × 10^5^ HEK293 cells were incorporated into chitosan-alginate hybrid hydrogels containing 4 µg hGLuc mRNA complexed with transfection reagent (TR). Furthermore, hydrogels without mRNA and TR or without TR were generated. PrestoBlue cell viability assay was performed 24, 48, and 72 h after incubation of hydrogels at 37 °C. 1.5 × 10^5^ HEK293 cells cultivated in 12-well plates were analyzed as positive controls. The viability of cells without hydrogel was set to 100% and the viability of cells in hydrogels was expressed relative to cells without hydrogel. Data are shown as mean ± SEM, (*n* = 3). Statistical differences were analyzed using one-way ANOVA with Bonferroni’s multiple comparisons test. (ns: non-significant).

To assess the influence of synthetic mRNA loading in chitosan-alginate hydrogels on cell viability, 1.5 × 10^5^ HEK293 cells were incorporated into the gels. The cell viability was measured 24, 48, and 72 h after the cultivation at 37 °C using PrestoBlue assay (Figure 4). The same number of cells was also seeded in one well of a 12-well plate without hydrogel. No statistically significant differences in cell viability were detected in hydrogels containing the hGLuc mRNA, transfection reagent, or only OptiMEM compared to the adherent growing cells without hydrogel. After 48 h of cultivation, a small reduction of cell viability to approximately 70% was detected in HEK293 cells cultivated in hydrogels compared to the adherent growing cells. This could probably be caused by adaptation differences between the cells encapsulated in hydrogels and adhesive growing cells on the cell culture plate. However, the decrease of cell viability was statistically not significant.

## 3. Discussion

Hydrogels are promising scaffolds for the delivery of drugs as well as for the cultivation of cells. In this study, different hydrogels were tested for the incorporation of synthetic mRNA. Injectable chitosan-alginate hybrid hydrogels loaded with synthetic modified hGLuc mRNA demonstrated the sustained release of the incorporated Cy3 hGLuc mRNA over 21 days. The incorporated hGLuc mRNA was bioactive and led to the production of hGLuc for up to 3 weeks. These results demonstrated that hydrogels containing cells can be additionally loaded with synthetic mRNA and applied for simultaneous delivery of mRNA to implanted cells for tissue engineering applications. Thereby, desired proteins can be produced by the incorporated cells over an extended period without the need of repeated administration of synthetic mRNA.

In order to analyze the release kinetic of synthetic mRNA from different hydrogels, alginate, chitosan, and chitosan-alginate hybrid hydrogels were generated. All hydrogels showed sustained release of mRNA over 21 days. The slowest release was detected in chitosan hydrogels and the fastest release was detected in alginate hydrogels. In comparison, the use of a chitosan-alginate hybrid hydrogel resulted in a faster release of mRNA than from the chitosan hydrogel and a slower release than from the alginate hydrogel. Chitosan is a positively charged polymer, thus, the interaction of the negatively charged mRNA with chitosan could lead to increased retention of the mRNA in hydrogels. In contrast, alginate is a negatively charged polymer and due to electrostatic repulsive forces, the synthetic mRNA could be released much faster from alginate hydrogels than from chitosan hydrogels. Krebs et al. demonstrated that the release of siRNA was delayed after the addition of positively charged polymers, such as polyethylenimine (PEI) or chitosan, to the calcium crosslinked alginate hydrogels [41]. In a recent study, the addition of chitosan to an alginate-based hydrogel resulted also in slower release of negatively charged sphingosine-1-phospate compared to alginate hydrogels alone [56]. In another study, Ma and colleagues subcutaneously injected siRNA containing chitosan hydrogels in mice and they were able to detect the Cy5-labeled siRNA for up to 7 days compared to less than one day for siRNA alone [45].

The results of our study showed that the release kinetics of the synthetic mRNAs can be modulated by using different biomaterials e.g., with different charges. Furthermore, the production of hGLuc over 21 days by HEK293 cells incorporated into hGLuc mRNA containing chitosan-alginate hydrogels demonstrated that the incorporated hGLuc mRNA can be successfully uptaken into cells. The burst release and the uptake of synthetic mRNA after the first day of incubation could be the reason for the production of the highest hGLuc amount in HEK293 cells at the second day of incubation. After the second day, the production of hGLuc decreased over time. This could be related to the degradation processes, such as the degradation of hydrogels as well as possible degradation of complexed mRNA. Additionally, it was demonstrated that the generation of mRNA transfection complexes is required to efficiently deliver the mRNA into the cells and to continuously produce the desired protein in cells. Using transfection reagents, mRNA molecules can be protected from nucleases and thereby, the stability can be improved and the uptake into the cells can be increased [57]. In addition, cell viability measurements over 3 days revealed that the incorporation of synthetic hGLuc mRNA into chitosan-alginate hydrogels has no negative influence on the viability of cells.

## 4. Materials and Methods

### 4.1. In Vitro mRNA Synthesis

The plasmid pEX-A2 containing the hGLuc encoding sequence was produced by Eurofins Genomics (Ebersberg, Germany). The DNA template for the synthesis of hGLuc mRNA was generated by PCR and transcribed into mRNA using an in vitro transcription (IVT) reaction as previously described [58]. Briefly, the insert containing the coding sequence of hGLuc was amplified by PCR using the forward primer: 5′-TTGGACCCTCGTACAGAAGCTAATACG-3′ and reverse primer: 5′-T_120_CTTCCTACTCAGGCTTTATTCAAAGACCA-3′ (Ella Biotech, Martinsried, Germany) and the HotStar HiFidelity polymerase kit (Qiagen, Hilden, Germany) according to the manufacturer’s instructions. PCR product was purified using MinElute PCR purification kit (Qiagen). Subsequently, IVT reaction was performed using MEGAscript^®^ T7 kit (Ambion, Glasgow, Scotland, UK) according to manufacturer’s instructions. The IVT reaction mixture contained 7.5 mM ATP and 1.875 mM GTP, and to improve the stability and the translation of mRNA and to reduce the immunogenicity, UTP and CTP were completely substituted by 7.5 mM pseudoruridine-5′-triphosphate (pseudo-UTP) and 7.5 mM 5-methylcytidine-5′-triphosphate (5-methyl-CTP) (TriLink Biotech, San Diego, CA, USA). Furthermore, 2.5 mM 3′-0-Me-m7G(5′)ppp(5′)G RNA Cap Structure Analog (ARCA, New England Biolabs, Frankfurt, Germany) was used for 5′-end capping, and the mRNA was dephosphorylated using 5 U Antarctic phosphatase (New England Biolabs, Frankfurt am Main, Germany). Additionally, the IVT reaction mixture contained 40 U RiboLock RNAse inhibitor (Thermo Scientific, Waltham, MA, USA) to prevent mRNA degradation. After the IVT and dephosphorylation, the mRNA was purified using RNeasy kit (Qiagen) and nuclease-free water was used for the elution of mRNA. The purity and specific length of generated PCR and mRNA product was analyzed using 1% agarose gel electrophoresis. Samples were run at 100 V for 45 min and the gel was stained with 1× GelRed™ (Biotium, Fremont, CA, USA) in 1× TBE buffer.

### 4.2. Fluorescent Labeling of mRNA

To determine the released mRNA from hydrogels, cyanine 3 (Cy3) labeled hGLuc mRNA was generated by Cu(I)-free dibenzocyclooctyne (DBCO) click chemistry. Therefore, the IVT mixture contained instead of 7.5 mM pseudo-UTP, 1.9 mM 5-azido-C_3_-UTP (Jena Bioscience, Jena, Germany) and 5.6 mM pseudo-UTP. Thereby, 25% of the total amount of pseudo-UTP was replaced by 5-azido-C_3_-UTP. Afterwards, 5-fold amount of DBCO-sulfo-Cy3 (Jena Bioscience) was added to the 5-azido-C3-UTP containing mRNA and the volume was adjusted with DPBS (w/o Ca^2+^/Mg^2+^) to 40 µL, vortexed for 10 s and incubated for 60 min at 37 °C. In order to remove the remaining unbound DBCO-sulfo-Cy3 molecules, the labeling mixture was purified using RNeasy kit according to manufacturer’s instructions.

### 4.3. Generation of mRNA Transfection Complexes and Incorporation into Hydrogels

Transfection complexes were generated by incubation of 4 µg hGLuc mRNA with 4 µL of the transfection reagent from the GenaxxoFect transfection kit and 8 µL of the dilution buffer (Genaxxon Bioscience, Ulm, Germany) for 15 min at room temperature (RT). Subsequently, the generated transfection complexes were filled with nuclease-free water up to 30 µL and added to the hydrogels, mixed, and incubated for 30 min at 37 °C to achieve complete gelation of hydrogels. Hydrogels with only transfection reagent or nuclease-free water were also prepared as negative controls.

### 4.4. Preparation of Hydrogels

#### 4.4.1. Alginate Hydrogels

A solution containing 3% (*w*/*v*) alginate was prepared by gradual addition of 300 mg alginate (Sigma-Aldrich, St. Louis, MO, USA) to 10 mL DPBS. After 1 h of constant stirring at 300 rpm and RT, alginate was completely dissolved and sterilized using UV light for 30 min. An aqueous solution containing 100 mM CaCl_2_ was prepared and filtered through a 0.22 µm sterile filter. Afterwards, CaCl_2_ solution was diluted in DPBS to 49 mM. For the incorporation of mRNA into the hydrogels, 88.7 µL of 3% alginate solution was added to 30 µL mRNA transfection complex mixture and mixed thoroughly. After adding 81.3 µL of 49 mM CaCl_2_ solution to the mRNA containing alginate solution, hydrogels consisting of 1.3% alginate and 19.9 mM CaCl_2_ were obtained.

#### 4.4.2. Chitosan Hydrogels

Chitosan with a deacetylation degree of 75–85% was purchased from Sigma-Aldrich and autoclaved. To obtain a solution with 3% (*w*/*v*) chitosan, 300 mg of the sterilized chitosan was dissolved in 10 mL DPBS containing 0.12 M hydrochloric acid. Chitosan was completely dissolved by initial stirring at 100 rpm for 30 min at RT and the following stirring for 30 min at 4 °C. Thermosensitive hydrogels were then produced by dropwise adding of 3.3 mL 20% sterile-filtered cold GP (Sigma-Aldrich, St. Louis, MO, USA) solution in DPBS. After stirring for another 60 min at about 300–350 rpm and 4 °C, the transfection complexes (30 µL) were added to the hydrogel (170 µL). The resulting final concentration was 1.9% chitosan and 4.2% GP.

#### 4.4.3. Chitosan-Alginate Hybrid Hydrogel

To generate the chitosan-alginate hybrid hydrogels, 3% (*w*/*v*) chitosan solution was prepared as described above. The chitosan solution (10 mL) was mixed at 4 °C with 4.4 mL 20% GP solution by stirring at 600 rpm for 5–10 s and then at 100 rpm for 1 h. Subsequently, 4.14 mL of 3% alginate solution was added and stirred at 350 rpm for 1 h. After mixing the gel (170 µL) with the mRNA transfection complexes (30 µL), the final concentration was 1.4% chitosan, 0.6% alginate, and 4.0% GP.

#### 4.4.4. Detection of Released Cy3-Labeled hGLuc mRNA from Hydrogels

To perform release experiments, 200 µL of each hydrogel, containing 30 µL of the transfection complex mixture (4 µg of Cy3-labeled mRNA in 18 µL nuclease-free water, 4 µL of GenaxxoFect transfection reagent, and 8 µL of dilution buffer) was generated per well of 48-well plates. Negative controls were prepared by adding the required amount of nuclease-free water, transfection reagent and dilution buffer or only nuclease-free water and dilution buffer. After complete gelation of the hydrogels, 150 µL DPBS was added to each well and incubated at 37 °C. The supernatant was collected after 4 h, 1, 2, 3, 7, 10, 14, and 21 days. After each time point, 150 µL fresh DPBS was carefully pipetted onto the hydrogels and further incubated. The mRNA, released from the hydrogels, was analyzed in duplicates (2 × 50 µL) using the Mithras LB 940 Multimode Microplate Reader (Berthold Technologies, Bad Wildbad, Germany). Fluorescence intensity was measured using the excitation/emission wavelength of 495 nm/540 nm. To quantify the released mRNA amount, a standard curve of Cy3-labeled mRNA with concentrations ranging from 31.25 ng to 1000 ng per 50 µL DPBS was used.

### 4.5. Rheological Characterization of Chitosan-Alginate Hybrid Hydrogels Containing hGLuc mRNA

To assess hydrogel characteristics and the stability of hydrogels over several days in vitro, hydrogels were produced as mentioned above and 200 µL of hydrogel without and with 4 µg mRNA was transferred into 1.5 mL reaction tube and allowed to solidify for 1 h at 37 °C. Then, the first rheological measurement (day 0) was performed after removing air bubbles by centrifugation at 100× *g* for 1 min. Next, 0.5 mL DPBS was added to the gels followed by further incubation at 37 °C. The rheological characterization of the hydrogels was then performed after 1, 7, 14, and 21 days of incubation at 37 °C using a squeeze-flow rheometer, called piezo axial vibrator (PAV) [59]. The storage G′ (elastic) and loss storage G″ (viscous) moduli of chitosan-alginate hydrogels were assessed by oscillatory squeeze-flow measurements using a gap size of 100 µm. Hydrogels of 200 µL were placed on the bottom plate of the PAV and the lid was carefully fixed with a defined pressure. The experiments were performed at RT with a frequency scan of 10–100 Hz.

### 4.6. Cultivation of HEK293 Cells

HEK293 cells were cultivated in DMEM with high glucose and l-glutamine supplemented with 10% FBS and 1% penicillin/streptomycin (all from Life Technologies, Darmstadt, Germany). Cells were kept at 37 °C with 5% CO_2_ and the medium was changed every 3 days. Cells were rinsed with 1 mL DPBS and detached using 0.04% trypsin/0.03% EDTA and 0.05% trypsin inhibitor in 0.1% BSA (both from PromoCell, Heidelberg, Germany). After centrifugation for 5 min at 300× *g*, the cell pellet was resuspended in culture medium.

### 4.7. Influence of hGLuc mRNA Loading in Chitosan-Alginate Hydrogels on Cell Viability

Chitosan-alginate hydrogels containing 1.5 × 10^5^ HEK293 cells and 4 µg hGLuc mRNA were generated and transferred into one well of a 12-well plate. Then, hydrogels were incubated at 37 °C with 1 mL cell culture medium. The cell viability was measured after 24, 48, and 72 h using PrestoBlue^®^ assay (Invitrogen, Carlsbad, CA, USA). Therefore, 110 µL PrestoBlue^®^ cell viability reagent was added to 1 mL cell culture medium per well and incubated for 1.5 h at 37 °C. The fluorescence intensity of 50 µL supernatant was measured in triplicates at excitation wavelength of 530 nm and emission wavelength of 600 nm using multimode microplate reader (Mithras LB 940, Berthold Technologies).

### 4.8. Analyses of the Bioactivity of Synthetic hGLuc mRNA Incorporated in Chitosan-Alginate Hydrogels

To analyze the transfectability and the functionality of the incorporated hGLuc mRNA in chitosan-alginate hydrogels, 1.5 × 10^5^ HEK293 cells were added in 100 µL cell culture medium to 500 µL chitosan-alginate hydrogels containing 4 µg hGLuc mRNA and carefully mixed. Hydrogels were then transferred into 12-well plates. After thermal gelation for about 30 min at 37 °C, 1 mL cell culture medium was added to the gels and incubated at 37 °C with 5% CO_2_. The production of secreted hGLuc by HEK293 cells was determined over 21 days in medium using luciferase assay.

### 4.9. Luciferase Assay

To measure the luciferase activity in the supernatants, DPBS containing 20 µg/mL coelenterazine (Carl Roth, Karlsruhe, Germany) was prepared. During the measurement, 100 µL coelenterazine solution was automatically injected into each well of a 96-well plate containing 40 µL supernatant in triplicates. The resulting bioluminescence was immediately detected as relative light units (RLU) using multimode microplate reader (Mithras LB 940).

### 4.10. Statistical Analysis

Data are shown as mean ± standard deviation (SD) or standard error of mean (SEM). One or two-way repeated measures analysis of variance (ANOVA) followed by Bonferroni’s multiple comparison test was performed to compare the means. All statistical analyses were performed double-tailed using GraphPad Prism version 6.01 (GraphPad Software, La Jolla, CA, USA). Differences of *p* < 0.05 were considered significant.

## 5. Conclusions

**Figure 5:**
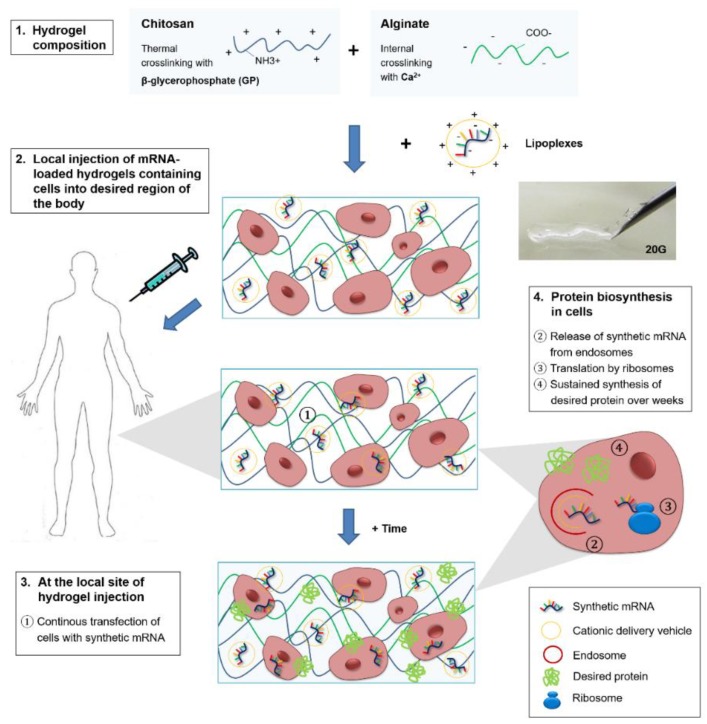
Schematic representation of the future clinical application of synthetic mRNA-loaded injectable chitosan-alginate hybrid hydrogels for local and sustained delivery of synthetic mRNAs. (**1**) The chitosan-alginate hybrid hydrogel is made of opposite charged polymers chitosan and alginate; (**2**) After the addition of mRNA containing transfection complexes (lipoplexes) and cells, the obtained hydrogels are injectable through a 20 G needle. Due to the use of β-glycerophosphate (GP) for thermal activated crosslinking and an internal crosslinking of alginate by Ca^2+^, the gel can solidify inside patient’s body at a desired position; (**3**) At the local site of injection, the lipoplexes can lead to the continuous transfection of cells with synthetic mRNA; (**4**) After the release of mRNA from endosomes into the cytosol, the ribosomal translation of mRNA into proteins starts and results in sustained synthesis of desired proteins.

This study confirmed the potency of chitosan-alginate hydrogels for prolonged delivery of mRNA transfection complexes until 21 days in vitro. Above all, these promising results demonstrated for the first time that hydrogels can be loaded with synthetic mRNAs for tissue engineering applications. A schematic representation of the future clinical application of injectable chitosan-alginate hybrid hydrogels loaded with synthetic mRNAs is displayed in Figure 5. The mRNA-loaded hydrogels could be applied for local and continuous synthetic mRNA delivery to cells over weeks for diverse approaches, e.g., to produce growth or differentiation factors to improve tissue regeneration.

## Supplementary Materials

Supplementary materials can be found at http://www.mdpi.com/1422-0067/19/5/1313/s1.
