# Supplementary material for: Incorporation of Synthetic mRNA in Injectable Chitosan-Alginate Hybrid Hydrogels for Local and Sustained Expression of Exogenous Proteins in Cells
Source: Int J Mol Sci. 2018 Apr 27;19(5):1313. doi: 10.3390/ijms19051313 (PMC5983784; doi:10.3390/ijms19051313)
Supplement: Supplementary file 1 [file ijms-19-01313-s001.pdf]

Supplementary Figure 1

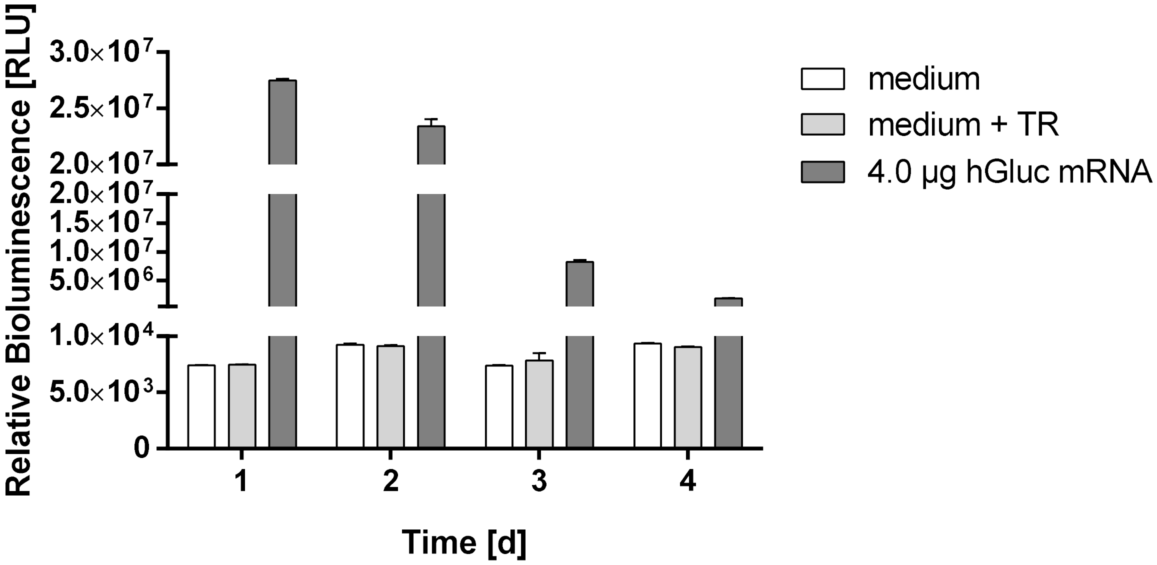

Figure S1. Luciferase activity after the transfection of HEK923 cells with synthetic hGLuc mRNA. TR: Transfection Reagent
